# Development and Validation of a Novel Model to Predict Liver Histopathology in Patients with Chronic Hepatitis B

**DOI:** 10.1155/2019/1621627

**Published:** 2019-02-27

**Authors:** Hongying Guo, Beidi Zhu, Shu Li, Jing Li, Zhiqing Shen, Yijuan Zheng, Weidong Zhao, Dan Tan, Jingwen Wu, Xueyun Zhang, Qirong Jiang, Xun Qi, Richeng Mao, Xueping Yu, Zhijun Su, Jiming Zhang

**Affiliations:** ^1^Department of Infectious Diseases, Huashan Hospital, Fudan University, Shanghai 200040, China; ^2^Department of Severe Hepatitis, Shanghai Public Health Clinical Center, Fudan University, Shanghai 200040, China; ^3^Department of Nutrition, First Hospital of Quanzhou, Fujian Medical University, Quanzhou 362000, China; ^4^Department of Infectious Diseases, First Hospital of Quanzhou, Fujian Medical University, Quanzhou 362000, China; ^5^Department of Laboratory Medicine, Clinical Medicine College, Dali University, Dali 671000, China

## Abstract

It is still vague for chronic hepatitis B (CHB) patients with normal or mildly increasing alanine aminotransferase (ALT) level to undergo antiviral treatment or not. The purpose of our study was to establish a noninvasive model based on routine blood test to predict liver histopathology for antiviral therapy. This retrospective study enrolled 258 CHB patients with liver biopsy from the First Hospital of Quanzhou (training cohort, n=126) and Huashan Hospital (validation cohort, n=132). Histologic grading of necroinflammation (G) and liver fibrosis (S) was performed according to the Scheuer scoring system. A novel model, ATPI, including aspartate aminotransferase (AST), total bilirubin (TBil), and platelets (PLT), was developed in training cohort. The area under ROC curves (AUC) of ATPI for predicting antiviral therapy indication was 0.83 in training cohort and was 0.88 in the validation cohort, respectively. Similarly, ATPI also displayed the highest AUC in predicting antiviral therapy indication in CHB patients with normal or mildly increasing ALT level. In conclusion, ATPI is a novel independent model to predict liver histopathology for antiviral therapy in CHB patients with normal and mildly increased ALT levels.

## 1. Introduction

Hepatitis B virus (HBV) infection is a global public health problem that can induce liver histopathology and may subsequently lead to the development of liver cirrhosis (LC) and hepatocellular carcinoma (HCC) [1]. Antiviral therapy is the first line for chronic hepatitis B (CHB) patients to prevent disease progression [2, 3].

CHB patients with elevated ALT levels >2 upper limit of normal (ULN) should be considered to initiate antiviral treatment. However, ALT level is affected by many factors, such as infection, alcohol, drugs, and hemolytic heart disease. In some patients, ALT level is not correlated with hepatitis B infection, significant liver necroinflammation, or fibrosis. Meanwhile, patients with persistently normal or mild ALT increase (<2ULN) may exist significant necroinflammation and fibrosis. The degree of liver necroinflammation or fibrosis is essential for decisions on antiviral treatment [4] and ALT is insufficient to predict it faithfully. Liver biopsy (LB), the golden standard to evaluate liver necroinflammation or fibrosis [5], is not widely used in clinic [6]. FibroScan is also less readily available, especially in resource-limited settings, and less useful in liver necroinflammation diagnosis [7, 8]. A new noninvasive method to predict liver necroinflammation or fibrosis is essential for antivirus therapy evaluation to promote precise treatment.

Recently, serum biomarkers, including aspartate aminotransferase (AST) to platelet (PLT) ratio index (APRI) [9], Fib-4 (based on age, ALT, AST, and PLT) [10], and *γ*-glutamyl transpeptidase (GGT) to platelet (GPR) [11], have been reported to effectively and accurately predict significant fibrosis and cirrhosis in patients with CHB and/or hepatitis C. However, these biomarkers were mainly based on patients with hepatitis C or with HCV/HIV coinfection and might produce inconsistent results in CHB patients. And the clinical heterogeneity postulates the application of these biomarkers in CHB patients. Recently, several models in CHB patients have been reported to predict liver fibrosis or cirrhosis but not liver histopathology for commencing antiviral therapy evaluation. And one noninvasive model, to predict liver histopathology for commencing antiviral therapy, was constructed in HBeAg-positive CHB patients with ALT ⩽2 ULN [12], which is not representative of the general CHB patients in China.

This study aims to develop a novel method predictive index based on routine blood tests to predict liver histopathology for commencing antiviral therapy in Chinese CHB patients. We compared diagnostic accuracy of noninvasive biomarkers in a Chinese CHB cohort, then to develop and validate a novel predictive ATPI index based on routine blood tests, including AST, PLT, and total bilirubin (TBil), to predict liver histopathology for commencing antiviral therapy in patients with CHB.

## 2. Materials and Methods

### 2.1. Patients

**Figure 1:**
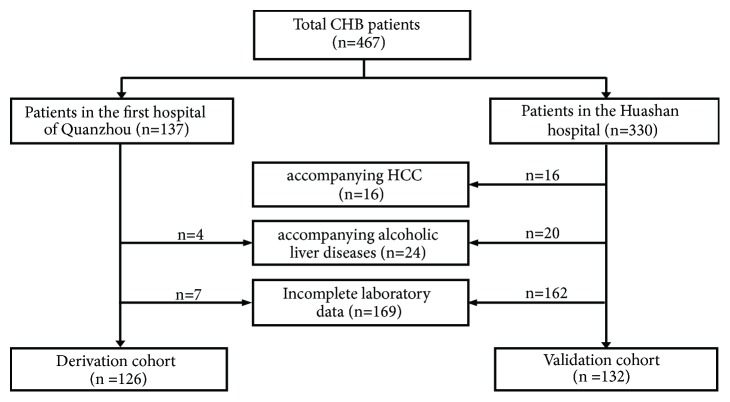
Flow diagram of the study design and recruitment of study subjects. Patients with chronic HBV infection (n=467) from the First Hospital of Quanzhou (n=137, Quanzhou, China) and Huashan Hospital (n=330, Shanghai, China) were evaluated. A total of 258 patients with liver biopsy at baseline were included in this study. Histologic grading of necroinflammation (G) and staging of liver fibrosis (S) were performed according to the Scheuer scoring system.

We conducted an analysis of a retrospective cohort study at First Hospital of Quanzhou, Fujian Medical University (training cohort) from 1994 to 2008, and an independent cohort study at Huashan Hospital, Fudan University (validation cohort), from 2006 to 2016 using the same criteria. All patients showed evidence of hepatitis B surface antigen (HBsAg) that persisted for >6 months, which defined as CHB [3, 4, 13]. The exclusion criteria are that (1) the patients coinfected with viral hepatitis (HAV, HCV, HDV, and HIV) (2) and patients had heart disease, thyroid disease, and kidney disease and had antivirus therapy. A total of 467 CHB patients were enrolled in the study.  Figure 1 summarizes the flow diagram of the study population. Two hundred and nine patients were excluded due to companying HCC (n=16) and alcoholic liver diseases (n=24) and insufficient laboratory data (n=169). The final study population consisting of 258 patients was divided into a training group (Quanzhou cohort, n=126) and a validation group (Shanghai cohort, n=132). The demographic, biochemical, and histological characteristics of all CHB patients in two groups are shown in Table 1. All trials had been approved by the Ethics Committee of First Hospital of Quanzhou and Huashan Hospital.

### 2.2. Data Collection

Patient's demographics and laboratory parameters were recorded within 7 days following LB. Serum albumin (ALB), globulin (GLB), total bilirubin (TBIL), alanine transaminase (ALT), aspartate aminotransferase (AST), alkaline phosphatase (ALP), and gamma-glutamyl transpeptidase (GGT) levels were measured by an automatic biochemical analyzer (Hitachi 7600P, Hitachi, Japan) for Shanghai set and automatic biochemical analyzer (Beckman LX-20; Beckman, Brea, CA) for Quanzhou set. HBsAg, anti-HBs, hepatitis B e Antigen (HBeAg), anti-HBe, anti-HBc, anti-HAV, and anti-HCV were analyzed by a system (ARCHITECT® i2000SR, Abbott, USA) for Shanghai and Quanzhou set. Serum HBV DNA was detected using commercially available kits on Light Cycler 480 Real-time PCR system (Roche, Basel, Switzerland) for Shanghai set and on a PE 9700 Thermal Cycler (Perkin Elmer, Boston, MA) for Quanzhou set according to the manufacturer's instructions. The white blood count (WBC), granulocyte ratio (GR), lymphocyte ratio, red blood count (RBC), hemoglobin (Hgb), and platelet count (PLT) levels were analyzed by automatic blood cell analyzer (Coulter LH 750, Beckman, Fullerton, CA, USA) for Shanghai and Quanzhou set.

### 2.3. Liver Biopsy

Percutaneous LB under ultrasound guidance was performed using disposable needle (Manan Super-Core, Medical Device Technologies co., LTD, Gainesville, Florida, USA) for training cohort and using a 16G needles (MAX-CORE® MC1616, BARD® Peripheral Vascular, Inc., USA) for validation cohort. Liver samples with less than a minimum length of 1.5 cm were poor biopsy samples and were excluded from the study. The specimens were formalin-fixed, paraffin-embedded, and stained with H&E for histological analysis. Histologic grading of necroinflammation (G0-G4) and staging of liver fibrosis (S0-S4) were performed according to the Scheuer scoring system [14] by specialized pathologists. G*⩾*2 and S*⩾*2 were considered to indicate moderate/severe inflammation and moderate/severe fibrosis, respectively. According to the AASLD [3], EASL [4], and APASL [13] practice guidelines, patients with necroinflammation ≥G2 or fibrosis ≥S2 need antiviral therapy.

### 2.4. Noninvasive Methods and Calculation Formulae

GPR, Fib-4, AST-to-platelet ratio index (APRI), and HBeAg (+) model were calculated as previously described: GPR= GGT (IU/L) × 100/PLT (10^9^/L); Fib-4= Age (year) × AST (IU/L) / (PLT (× 10^9^/L) × ALT (IU/L)^1/2^); APRI= AST (IU/L) × 100/PLT (× 10^9^/L); and HBeAg (+) model= 5.956 *∗* log10 (AST) - 2.612 *∗* log10(HBsAg) - 0.016 *∗* PLT (× 10^9^/L) - 0.15 *∗* ALB(g/L) + 9.544.

### 2.5. Statistical Analysis

The data were analyzed using SPSS 13.0 (SPSS Inc., Chicago, IL, USA) and MedCalc® 15.8 (MedCalc Software BVBA, Ostend, Belgium). The data are expressed as the median (interquartile ranges). Differences between groups were compared using Mann-Whitney nonparametric U test for continuous variables, and using chi-square test for categorical variables. Correlation was analyzed by the Spearman's rank correlation coefficient. Univariate and multivariate logistic regression were used to develop a new model for predicting G*⩾*2 or S*⩾*2. The receiver operating characteristic (ROC) curve was used to evaluate the diagnostic performance of this new model and other indexes, and the results were expressed as a hazard ratio with 95% confidence interval (95% CI). The sensitivity, specificity, positive predictive value (PPV), and negative predictive value (NPV) were calculated to explore the best cutoff value. P-values<0.05 were considered as statistically significant.

## 3. Results

### 3.1. Predictors and Regression Models

In the training cohort, patients were divided into two sets: nonantiviral set (G<2 and S<2) and antiviral set (G*⩾*2 or S*⩾*2). Univariate analysis revealed that PLT (P=0.017), TBil (P=0.002), ALT (P=0.006), AST (P=0.002), GGT (P=0.008), and HBeAg (P=0.018) of the 18 variables were independent predictive factors for antiviral therapeutic indications and significantly different in this two group (Table 2). There were no differences among the other indicators (all P>0.05).

**Figure 2:**
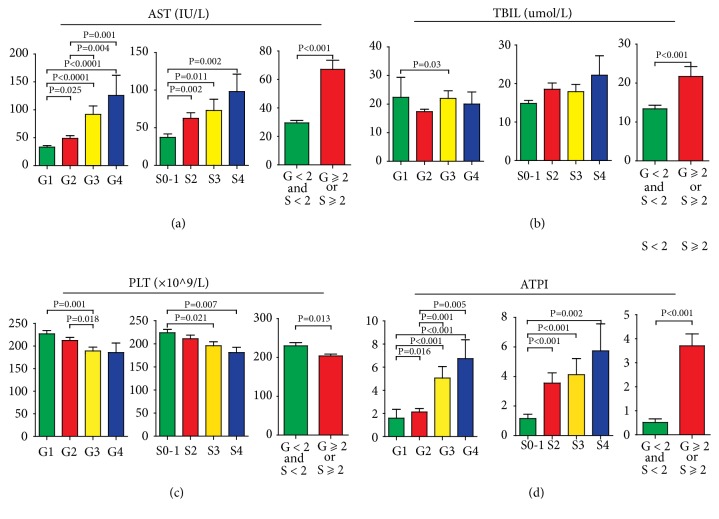
Histogram of (a) aspartate aminotransferase (AST), (b) total bilirubin (TBil), (c) platelets (PLT), and (d) ATPI model according to Scheuer scoring system in the training cohort.

Step-forward multiple regression analysis all revealed that TBil (P=0.035), AST (P=0.01), and PLT (P=0.037) were independently correlated with antiviral therapeutic indications. The final multiple regression model incorporating TBil, AST and PLT was ATPI model= 0.054×AST (g/L) + 0.09×TBil (*μ*mol/L) – 0.008× PLT (10^9^/L) – 0.366. The new ATPI model and its component factors (TBIL and AST) progressively increased with the stage ascending of liver necroinflammation or fibrosis (Figures 2(a), 2(b), and 2(d)), in contrast to PLT levels (Figure 2(c)). Furthermore, ATPI model was strongly positively associated with liver inflammation (r=0.472, P<0.001) and liver fibrosis (r=0.417, P<0.001). The median value for ATPI model in nonantiviral group (0.618) was significantly lower than that in antiviral group (2.128) (Figure 2). Therefore, the new ATPI model based on TBil, AST, and PLT levels may be a good independent indicator to reflect the degree of liver necroinflammation and fibrosis.

### 3.2. Comparisons of ROC between ATPI and Other Established Noninvasive Models

In the training cohort, necroinflammation stage according to histopathology was as follows: 0 patients (0.0%) were G0, 34(27.0%) were G1, 56(44.4%) were G2, 28(22.2%) were G3, and 8(6.3%) were G4. And 6 patients were (4.8%) in S0, 39(31.0%) in S1, 40(31.7%) in S2, 30(23.8%) in S3, and 11(8.7%) in S4 based on the liver fibrosis stage.

**Figure 3:**
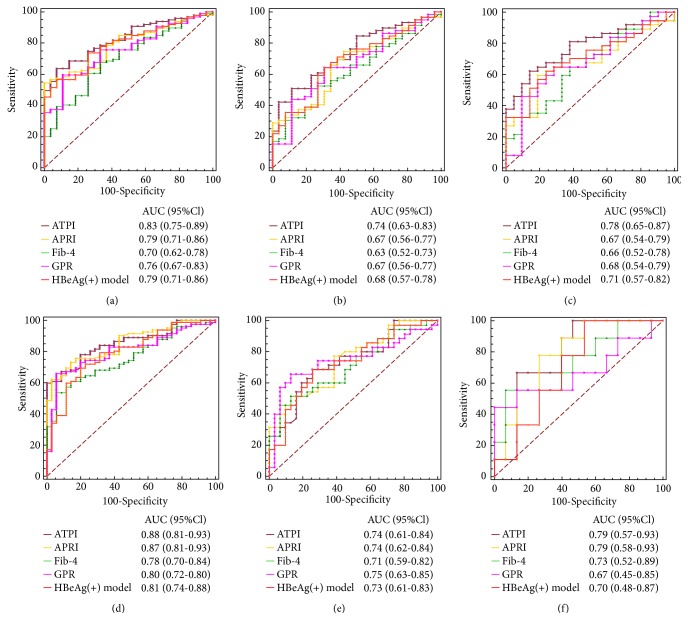
ROC of noninvasive biomarkers, including new ATPI model, AST-to-platelet ratio index (APRI), Fib-4 (based on age, ALT, AST, and platelet count), *γ*-glutamyl transpeptidase (GGT) to platelet (GPR), and HBeAg(+) model for predicting liver histopathology for commencing antiviral therapy in total patients from (a) training cohort and (d) validation cohort; patients with ALT≤2 upper limit of normal (ULN) from (b) training cohort and (e) validation cohort; patients with ALT≤ 1 ULN from (c) training cohort and (f) validation cohort.

For predicting antiviral therapeutic indications, the AUC of ATPI (0.83, 95% CI 0.75-0.89) was significantly higher than that of Fib-4 (0.70, 95% CI 0.62-0.78, P=0.04), but slightly higher than APRI (0.79, 95% CI 0.71-0.86h, P=0.15), GPR (0.76, 95% CI 0.67-0.83, P=0.13), and HBeAg (+) model (0.79, 95% CI 0.71-0.86, P=0.33) (Table 3; Figure 3(a)). For predicting the indications of antiviral therapy, the sensitivity and specificity were 63.64% and 92.59%, with a best cutoff value 1.53.

### 3.3. Validation Set in Shanghai Cohort

In the validation cohort, necroinflammation stage according to histopathology was as follows: 17(12.9%) in G0, 28(21.2%) in G1, 49(37.1%) in G2, 36(27.3%) in G3, and 2(1.5%) in G4 (Table 1). And 27(20.5%) were S0, 29 (22.0%) were S1, 45 (34.1%) were S2, 17 (12.9%) were S3, and 14 (10.6%) were S4 based on the liver fibrosis stage.

For prediction of antiviral therapeutic indications, the AUC of ATPI (0.88, 95% CI 0.81-0.93) was significantly higher than that of Fib-4 (0.78, 95% CI 0.70-0.84, P=0.02), while slightly higher than that of APRI (0.87, 95% CI 0.81-0.93, P=0.93), GPR (0.80, 95% CI 0.72-0.80, P=0.24) and HBeAg (+) model (0.81, 95% CI 0.74-0.88, P=0.17) (Table 3, Figure 3(d)). Also, the best cutoff value of ATPI for antiviral therapeutic indication was 1.53. There were 69 patients presented with ATPI > 1.53, of whom 66 (95.65%) had antiviral therapeutic indications.

### 3.4. Diagnostic Accuracy of ATPI in Patients with Normal and Mildly Increasing ALT Level

In clinic, many CHB patients with normal or mildly increasing ALT level already have severe liver necroinflammation or even progress to cirrhosis or HCC. In the training cohort (n=126), 58 patients (46.03%) had normal ALT levels and 86 patients (68.25%) had mildly increasing ALT level, of which 37 (65.52%) and 59 (68.60%) patients had significantly necroinflammation, 34 (58.62%), and 47 (55.29%) patients had significantly liver fibrosis, respectively. In the validation cohort (n=132), 25 patients (18.94%) had normal ALT level and 86 patients (65.15%) had mildly increasing ALT level, of which 6 (24.00%) and 59 (68.60%) patients had significant necroinflammation, and 7 (28.00%) and 35 (40.70%) patients had significantly liver fibrosis, respectively. Therefore, we determined whether ATPI could be used to separate antiviral from nonantiviral set in patients with normal ALT (⩽1 ULN) and mildly increasing ALT (⩽2 ULN).

ATPI also perform good predict value in patients with normal and mildly increasing ATL. The AUC of ATPI (95% CI) were slightly higher than APRI (95% CI), Fig-4 (95% CI), GPR (95% CI), and HBeAg (+) model (95% CI) in training and validation cohort in patients with ALT ⩽2 ULN (Table 4; Figures 3(b) and 3(e)). Similar results also were observed in patients with ALT ⩽1 ULN (Table 4, Figures 3(c) and 3(f)). These data suggest that ATPI is more specific than other models in predicting antiviral therapy indication in patients with normal or mildly increasing ALT level.

## 4. Discussion

In this study, a noninvasive model (named as ATPI, composed of AST, TBil, and PLT) was established to predict antiviral therapy indication in CHB patients. Finally, 95.45% (63/66) patients in the training cohort, 95.65% (66/69) in the validation cohort, in other words 95.56% (129/135) in the entire cohort can be properly evaluated for antivirus therapy, with a best cutoff value 1.53. Therefore, the ATPI might be a potential efficient noninvasive model to determine whether to initiate antiviral treatment in CHB patients.

Many noninvasive models have been established recently to estimate liver fibrosis or cirrhosis with high accuracies, indicating the urgent requirement in clinic. However, few of them were further identified as good predictors for antivirus therapy indication due to Various known and unknown causes. APRI was first proposed by Wai CT et al. in 2003 in patients with chronic hepatitis C and written in several authoritative clinical practice guidelines. However, it had limited diagnostic accuracy in CHB cohort [9]. Also, Fib-4 can accurately differentiate mild to moderate fibrosis from fibrosis and cirrhosis in patients coinfected with HIV/HCV [10]. By contrast, GPR was firstly considered as a more accurate marker than APRI and Fib-4 to stage liver fibrosis in patients with chronic HBV infection in West Africa [11]. In our another cohort, GPR also had been demonstrated with relatively higher accuracy in diagnosing liver fibrosis and cirrhosis compared to other established noninvasive [15]. Therefore, it is necessary to know whether these noninvasive models are adapted to predict antiviral therapy indication. In this study, we summarized the diagnostic accuracy of the above noninvasive markers and found that APRI, GPR, and Fib-4 also could be applied to predict liver histopathology for initiating antiviral therapy, although they had modest predict accuracy.

To improve the prediction of antiviral therapy indication, a novel model, named as ATPI, was developed with TBil, AST and PLT count, which were all reported to be related with liver histopathology [9–11, 16]. In our training cohort, ATPI displayed the highest AUC value in predicting antivirus therapy indication, although there were no significantly difference between ATPI and APRI or GPR or HBeAg (+) model, the sensitivity and specificity of which in predicting antivirus therapy indication were 63.64% and 92.59%, with a best cutoff value 1.53. The similar results were seen in the validation cohort.

We also determined whether ATPI could be used for differentiating antiviral or nonantiviral therapy set in patients with normal and mildly increased ATL. In training cohort, more than 50% patients with normal or mildly increased ALT level had significantly necroinflammation and fibrosis, which similar with the results of previous studies [17]. Similarly, ATPI also perform as greatly as in total patients from training and validation cohort, displaying the highest AUC in predicting antivirus therapy indication in patients with normal or mildly increasing ALT level.

There are some limitations in the present study. First, there were demographic, biochemical, and histological differences between these two cohorts, which may lead to different results in two groups. Second, we were not able to consider other laboratory variables of potential interest in CHB, such as HBV virology index (HBsAg, HBeAg, and anti-HBc) due to the data which were also not always fully complete.

## 5. Conclusions

Our study showed that ATPI is a novel independent indicator for predicting antiviral therapy indication, especially in patients with normal and mildly increased ALT levels. By applying the predefined cutoffs, most patients can be correctly classified as needing antivirus therapy or not. Thus, ATPI is a good surrogate marker for LB to determine whether to use antiviral treatment.

## Figures and Tables

**Table 1:** Baseline characteristics of the study population in training cohort and in validation cohort.

Variables	Training cohort (n=126)	Validation cohort (n=132)	P
Male, sex, n (%)	90 (71.43)	106 (80.30)	0.095
Age, years	26.00 (18.00-39.00)	33.50 (24.30-49.10)	*<0.001*
HBeAg+, n (%)	123 (97.62)	89 (67.4)	*<0.001*
HBV DNA, lg copies/mL	3.72 (2.62-4.48)	7.62 (5.83-8.18)	*<0.001*
ALB, g/L	41.40 (35.70-46.13)	45.00 (43.00-47.00)	*<0.001*
GLB, g/L	29.90 (25.04-35.04)	31.00 (28.00-34.00)	*0.047*
TBil, *μ*mol/L	16.30 (9.07-29.83)	12.25 (9.73-16.10)	*<0.001*
ALT, IU/L	44.50 (20.00-261.10)	73.00 (45.00-125.75)	*<0.001*
AST, IU/L	37.00 (22.00-125.50)	41.00 (28.00-68.75)	0.281
GGT, IU/L	21.50 (11.00-102.60)	26.00 (15.50-52.00)	0.388
ALP, IU/L	70.50 (43.80-122.30)	77.00 (61.00-88.00)	0.066
PT, s	12.30 (11.20-13.56)	11.50 (10.90-12.10)	*<0.001*
WBC, 10^9^/L	6.15 (4.27-8.63)	5.39 (4.30-6.37)	*<0.001*
GR, %	55.75 (41.78-68.12)	57.95 (50.25-65.10)	0.097
Lym, %	35.30 (23.97-48.70)	31.20 (26.58-39.43)	*0.006*
RBC, 10^12^/L	4.76 (4.14-5.36)	4.89 (4.62-5.26)	*0.041*
Hgb, g/L	146.00 (123.40-163.00)	151.00 (140.00-159.00)	*0.012*
PLT, 10^9^/L	210.00 (141.70-265.70)	176.50 (144.00-215.25)	*<0.001*
Necroinflammation stage, n (%)			
G0/G1/G2/G3/G4	0 (0.0)/34 (27.0)/56 (44.4)/28 (22.2)/8 (6.3)	17 (12.9)/28 (21.2)/49 (37.1)/36 (27.3)/2 (1.5)	*<0.001*
Significant inflammation (G2–G4)	92 (73.02)	87 (65.9)	0.216
Fibrosis stage, n (%)			
S0/S1/S2/S3/S4	6 (4.8)/39 (31.0)/40 (31.7)/30 (23.8)/11 (8.7)	27 (20.5)/29 (22.0)/45 (34.1)/17 (12.9)/14 (10.6)	*0.001*
Significant fibrosis (S2-S4)	81 (64.29)	76 (57.6)	0.270

HBsAg, hepatitis B surface antigen; HBeAg, hepatitis B e antigen; GLB, globulin; ALB, albumin; TBil, total bilirubin; ALT, alanine aminotransferase; AST, aspartate aminotransferase; ALP, alkaline phosphatase; GGT, gamma-glutamyl transpeptidase; PT, prothrombin; WBC, white blood count; GR, granulocyte ratio; RBC, lymphocyte ratio, red blood count; Hgb, hemoglobin; PLT, platelet count. Continuous variables were expressed as the median (25th to 75th percentile).

**Table 2:** Univariate and multivariate analyses of the relationships between biomarker and antiviral therapy indication in the training cohort.

Variables	Univariate	P-value	Multivariate	P-value
PLT	0.989 (0.980-0.998)	*0.017*	0.992 (0.982-0.102)	*0.037*
TBil	1.140 (1.048-1.241)	*0.002*	1.094 (1.006-1.190)	*0.035*
ALT	1.031 (1.009-1.053)	*0.006*		
AST	1.064 (1.022-1.107)	*0.002*	1.055 (1.013-1.099)	*0.010*
GGT	1.051 (1.013-1.090)	*0.008*		
HBeAg	0.996 (0.993-0.999)	*0.018*		

HBsAg, hepatitis B surface antigen; HBeAg, hepatitis B e antigen; GLB, globulin; ALB, albumin; TBil, total bilirubin; ALT, alanine aminotransferase; AST, aspartate aminotransferase; ALP, alkaline phosphatase; GGT, gamma-glutamyl transpeptidase; PT, prothrombin; WBC, white blood count; GR, granulocyte ratio; RBC, lymphocyte ratio, red blood count; Hgb, hemoglobin; PLT, platelet count.

**Table 3:** Comparison of ATPI model with APRI, FIB-4, GPR, and HBeAg (+) model.

Models	Training cohort	Validation cohort
*ATPI*		
AUC (95%Cl)	0.83 (0.75-0.89)	0.88 (0.81-0.93)
Cut-off values	1.53	1.53
Sensitivity/specificity (%)	63.64/92.59	69.47/91.89
PPV/NPV (%)	89.57/71.80	89.55/75.06
*APRI*		
AUC (95%Cl)	0.79 (0.71-0.86)	0.87 (0.81-0.93)
Cut-off values	21.15	21.15
Sensitivity/specificity (%)	54.62/100.00	74.74/86.49
PPV/NPV (%)	100/68.79	84.69/77.40
*Fib-4*		
AUC (95%Cl)	0.70 (0.62-0.78)	0.78 (0.70-0.84)
Cut-off values	0.66	0.66
Sensitivity/specificity (%)	60.61/74.07	83.16/43.24
PPV/NPV (%)	70.04/65.28	59.43/71.97
*GPR*		
AUC (95%Cl)	0.76 (0.67-0.83)	0.80 (0.72-0.80)
Cut-off values	10.57	10.57
Sensitivity/specificity (%)	59.60/88.89	75.61/65.71
PPV/NPV (%)	84.29/68.75	68.80/72.93
*HBeAg (+) model*		
AUC (95%Cl)	0.79 (0.71-0.86)	0.81 (0.74-0.88)
Cut-off values	3.61	3.61
Sensitivity/specificity (%)	56.57/92.59	93.68/35.14
PPV/NPV (%)	88.42/68.07	59.09/84.76
*Comparison of AUC (P value)*		
ATPI vs. APRI	0.15	0.93
ATPI vs. Fib-4	*0.03*	*0.02*
ATPI vs. GPR	0.10	0.24
ATPI vs.HBeAg(+) model	0.33	0.17
APRI vs. Fib-4	0.09	*0.01*
APRI vs. GPR	0.44	0.23
APRI vs. HBeAg(+) model	0.89	0.17
Fib-4 vs. GPR	0.37	0.44
Fib-4 vs. HBeAg(+) model	0.09	0.48
GPR vs. HBeAg(+) model	0.48	0.84

**Table 4:** Diagnostic accuracy of these models in patients with normal or mildly increasing ALT level.

Models	Training cohort						Validation cohort					
*Patients with* *ALT ⩽2ULN*	*AUC (95%Cl)*	*Compare with*					*AUC (95%Cl)*	*Compare with*				
		ATPI	APRI	Fib-4	GPR	HBeAg (+) model		ATPI	APRI	Fib-4	GPR	HBeAg (+) model
ATPI	0.74 (0.63-0.83)	1					0.74 (0.61-0.84)	1				
APRI	0.67 (0.56-0.77)	0.09	1				0.74 (0.62-0.84)	0.90	1			
Fib-4	0.63 (0.52-0.73)	0.12	0.51	1			0.71 (0.59-0.82)	0.66	0.52	1		
GPR	0.67 (0.56-0.77)	0.24	0.95	0.62	1		0.75 (0.63-0.85)	0.88	0.93	0.59	1	
HBeAg (+) model	0.68 (0.57-0.78)	0.32	0.65	0.39	0.79	1	0.73 (0.61-0.83)	0.94	0.87	0.75	0.78	1
*Patients with* *ALT ⩽1 ULN*												
ATPI	0.78 (0.65-0.87)	1					0.79 (0.57-0.93)	1				
APRI	0.67 (0.54-0.79)	0.06	1				0.79 (0.58-0.93)	0.93	1			
Fib-4	0.66 (0.52-0.78)	0.15	0.79	1			0.73 (0.52-0.89)	0.64	0.47	1		
GPR	0.68 (0.54-0.79)	0.15	0.92	0.78	1		0.67 (0.45-0.85)	0.50	0.39	0.68	1	
HBeAg (+) model	0.71 (0.57-0.82)	0.32	0.51	0.46	0.75	1	0.70 (0.48-0.87)	0.54	0.42	0.80	0.82	1

## Data Availability

The data used to support the findings of this study are available from the corresponding author upon request.
